# On the Alkaloids of Hydrastis Canadensis

**Published:** 1862-10

**Authors:** Wm. S. Merrill

**Affiliations:** Cincinnati


### ON THE ALKALOIDS OF HYDRASTIS CANADENSIS.

By WM. S. MERRILL, A.M.

To the Editor of the American Journal of Pharmacy:—

I hoped to be able to attend the approaching meeting of the American Pharmaceutical Association, but find I shall not be able to leave home. I have therefore forwarded to you a small package of samples, chiefly products obtained from the Hydrastis, and request you will present them to the Association for their examination.

In a business letter to Prof. Parrish, some months ago, I briefly mentioned these. I was not publishing an analysis or writing an essay, but briefly stated the fuller results of our experiments, of which I had previously sent him some samples; and I expressed a willingness to have these statements published, as they contained some facts that I did not suppose had been previously announced. Under these circumstances, I certainly thought some portion of your criticism of the article quite uncalled for.

Our examination of the hydrastis was principally made last fall, and I then knew nothing of the analysis of Durand, further than stated in U. S. D., which concludes by saying, "He also discovered a peculiar nitrogenous crystallizable substance for which he proposes the provisional name 'Hydrastin,' until it shall be determined whether it be, as he suspects, an organic alkali." This is certainly very indefinite, and does not even make it certain which of the two alkaloid bases it was which he discovered.

On the 6th of Feb. last, when sending to Prof. Parrish some samples of xanthoxylum bark and other articles for the cabinet which the Pharmaciens of Philadelphia were getting up for exhibition at the World's Fair, I inclosed with them specimens of *Hydrastina* and *Mur. Hydrastia*, which we had obtained, and briefly mentioned their leading properties. These were placed in the College cabinet, and there, Mr. Editor, you had seen them so labelled, and refer to them in your note to the article of Dr. Mahla in the Journal of that month. You certainly must have forgotten this, when in your criticism of July, speaking of my report that, "the Hydrastis contains *two* distinct alkaloids, which we name Hydrastia and Hydrastina," you say "this assumption is to be discountenanced," because I did not acknowledge the analysis of Durand, and Mahla and Perrins, as previous discoveries. Mr. Durand had discovered but one of these bases, and that hypothetically, and Dr. Mahla's article, which had not then appeared, speaks of but one. The articles of J. Dyson Perrins indeed recognises two, but these were not published in London until the April and May following, and I had seen nothing of them until their appearance in the same number of your Journal with the extract of my letter to Prof. Parrish.

It is evident in regard to all of us that our investigations were entirely independent of the others, and thus far *Original*, whichever may be entitled to credit in point of time.

I certainly have no wish to arrogate to myself the merits of other men's skill or discoveries, although I may be remiss in keeping myself posted in regard to them. And lest I be again accused of any such injustice, I beg leave here to state, I have not conducted these recent experiments alone. Fifteen years ago, I first introduced to the medical profession, the Podophyllin, Macortin, Leptandrin, and some others of that class of agents known as the "Resinoids" or "Concentrated remedies," and set the ball rolling in that direction. But the extensive mercantile and manufacturing business in which, in company with my brother, I became engaged, has since so engrossed my attention that I have found no time for the careful labors of chemical investigation and analysis. We have long felt, however, that many of that class of agents must be greatly improved and perfected by accurate and scientific analysis, or else be rejected from the list of medicinal agents, and have fully appreciated the wide field opened in vegetable materia medica, which, whatever may be our ability, we had no time to cultivate. We have, therefore, employed as an assistant in our laboratory, Prof. H. D. Garrison, a chemist of no mean acquirements, and while he works under our direction, and a good portion of the particular experiments and the steps pursued are at my suggestion, the *manipulations* are almost all performed and the results obtained by him.*

*NOTE BY THE EDITOR.—It seems needful to again refer to the subject of Mr. Merrill's paper, in order that we shall be fairly understood, both by the author and reader. The question at issue relates to the discovery of the active principles of the root of Hydrastis canadensis. In our July number, we published an extract of a letter to Mr. Parrish, with the authority of the writer, Mr. Merrill, and accompanied the paper with a critical note. In that paper Mr. Merrill spoke of the two alkaloids in the hydrastis, as though he had discovered them, and applies names to them as though they had not been written of before, he giving the commercial hydrastin the name of Durand's alkaloid, hydrastia; and calling the latter hydrastina; and it was to what we believed to be a disposition to ignore the labors of others that we applied the terms "this assumption is to be discountenanced." Mr. Merrill's explanations tend to modify this belief.

In the present paper, Mr. Merrill says, "It is evident in regard to all of us that our investigations were entirely independent of the others, whichever may be entitled to credit in point of time." "I certainly have no wish to arrogate to myself the merits of other men's skill or discoveries, although I may be remiss in keeping myself posted in regard to them."

Now we will not grant the position, that Mr. Merrill discovered either of the alkaloids, much less both of them, in the proper sense of making a discovery; nor can we admit Mr. Merrill's alleged ignorance of the details of Durand's paper, as an argument in his favor, in view of his knowledge of its existence near at hand, as he himself admits. In 1856, when sending out a collection of the American Materia Medica to Mr. Jacob Bell, for the Museum of the Pharmaceutial Society, we wrote to Mr. Wayne, of Cincinnati, for certain specimens not easily obtainable here. In his reply to my letter, Mr. Wayne, under date Cincinnati, May 23, 1856, says, . . . "I have sent you among the lot of specimens sanguinarina, sulphate of sanguinarina *hydrastin*, and *hydrastia?* I think the last *has all claims to be called an alkaloid.* The

In commencing our investigations, therefore, one of the first articles we took up was the hydrastis; and we thought we had acquired a pretty accurate knowledge of its constitution, and had briefly reported the result, when Prof. Parrish, in his letter of March 8th, acknowledging the receipt of the specimens, says: "The Hydrastina, and Muritate of 'Hydrastia', (Berberina,) and the salt of Sanguinarina are very acceptable, and are placed in the College cabinet, and adds, 'I suppose you have seen the late analysis of a Chicago chemist, transferred to the American Journal of Pharmacy for the current month. The specimen is as white as it can be made, *much more so than your pet specimen*, [alluding to a specimen of Durand's hydrastia in our cabinet, presented to us in 1851, and which we had shown him on a previous occasion]. I have a new process for obtaining *hydrastin*, at least I have seen no mention of it. I treat the coarse powdered root in cold water in a percolator. To the infusion I add an acid, generally muriatic acid, which throws down the hydrastin as a yellow powder, along with a pectin-like substance. . . . If the precipitate be now treated with boiling alcohol, only the hydrastin is taken up and crystallizes out upon cooling. By this process much less alcohol is lost than in the process of the Eclectic Disp. [Hill's process] and a much finer looking product obtained."

In regard to hydrastia, Mr. Wayne further says: "After I obtain the *hydrastin* from the cold infusion by an acid, I add to the acid liquor an alkali which precipitates a pale yellow powder, which being dried and treated with ether or alcohol, the hydrastia is taken up, and, upon evaporation, it is deposited in colored crystals, which by repeated solution and crystallization are obtained colorless."

Dr. John King, in the Eclectic Dispensatory, says, page 489—edit. 1859—in speaking of hydrastin (muriate of berberina:) "This elegant and highly valuable article was introduced to the profession by Dr. H. H. Hill, of the firm of F. D. Hill & Co., wholesale druggists in Cincinnati," and the process adopted by that gentleman is given in detail at page 480. By reference to the *advertising* sheet of the Eclectic Medical Journal for January, 1853, page 4, we find the following note attached to the name 'hydrastin' in a list of preparations advertised by F. D. Hill & Co., of Cincinnati, viz.: "This article, introduced by us one year ago, is one of the finest extant amongst Botanic medicines. In fact it is the quinine of North America." Hence Mr. Hill did not produce his Hydrastin until a year after Durand wrote, because Durand's Thesis was written in the summer of 1850—presented to the College at the session '50–'51, and of course not published till after the spring commencement in the latter year. Mr. Wayne's process is also given in that work, and nothing is said in reference to Mr. Merrill in that connection, albeit he was largely consulted by Dr. King in the compilation of his work. Our own impression had been that Durand's hydrastia was the only alkaloid in the hydrastis, and that the *hydrastin* of the Eclectic Dispensatory was the alkaloid of Durand, disguised by the yellow coloring matter of that author, [since shown to be berberina by Dr. Mahla], and the reader will find in the Pharmaceutical Journal, vol. 16th, November, 1856, page 269, and the following note that accompanied the sample of Eclectic hydrastin then sent to London: "*Hydrastin.* A small specimen of this principle, which is used by the Eclectics as a remedy, &c., &c. I believe the yellow color is mainly due to the adherent yellow resin or coloring matter, just as piperine is colored by the resine of pepper;" previous reference has been made to Durand's discovery of the alkaloid on the same page. From this it will be seen that at that time neither Mr. Wayne nor whole subject of the proximate principles of the hydrastis requires further ventilation."

In fact, however, I have not seen the article of Dr. Mahla referred to, and the above word in parenthesis was the first intimation I had that the alkaloid I had named Hydrastia, was identical with berberina. The analysis of Mahla was not entirely satisfactory, and I immediately set about having this point further investigated.

From some brief experiments, too crude to be reported as a scientific analysis, I had a long time before satisfied myself ourself had a correct notion of the nature of hydrastin. Subsequently to this —August, 1857—Mr. H. A. Tilden, of New Lebanon, sent us some samples of vegetable principles, and among them two labelled as follows: "Hydrastin," in silky crystals, and "Hydrastina alkaloid," a yellow amorphous powder. At that time we supposed it also was Durand's alkaloid, with yellow coloring matter, in a pulverized condition, but now, on trial, it is soluable in water to a considerable extent, coloring it yellow, and the solution restores the color of reddened litmus, and it is probably berberina.

Mr. Merrill's allusion to the specimens sent to Mr. Parrish, therefore, have nothing to do in deciding the case, as pure specimens of the same had been in our private cabinet for five years previously, as will be seen above.

It will be observed that Mr. Merrill recedes from his position in his former paper, that hydrastin is not berberina, he having been misled by the impurity of his sample of berberina.

We also wish to remark on Mr. Merrill's views in regard to the naming of those principles. He calls Durand's alkaloid Hydrastina, and the berberina alkaloid Hydrastia. We protest against this suggestion. Durand distinctly claims for his alkaloid (see page 117, vol. 23d, of the Amer. Journ. Pharm.,) if his assertion of its alkaline nature is corroborated, the name of *hydrastia.* Now, as *it* is the only peculiar alkaloid in hydrastis, it is entitled to that name, 1st, because it was given to it by Durand in 1851, and 2d, because it is the proper derivative for an alkaloid from the name hydrastis. Mr. Merrill argues because berberina is more abundant in the hydrastis than in other sources, it should be called hydrastia. This will not do, because berberina is found in at least four distinct drugs, and Columbo has equal claims. He also suggests "Xanthia" from its color. This is a beautiful name, and might have been appropriately used at first, but it is not probable that this name will be adopted, as M. Buchner, Sr. and Son, in 1835, describes this alkaloid in great detail, and gave it its present name, (see Journ. de Pharm, vol. xxi. page 408.).

Lastly, in justice to Mr. Durand, we wish to remove the impression conveyed in the paper of Mr. Merrill, that Durand's description of his alkaloid was very indefinite. So far from this being the case, he describes three distinct processes for obtaining it, proves its alkaline reaction, its crystalline form, its saturating power, its precipitability by tannic acid and ammonia, its total destructability by heat, and its nitrogenous nature. The only reason he hesitated to call it an alkaloid was the fact of its salts not crystallizing. Now, on this point Mr. Merrill says, it forms salts, "that are very soluable, and difficultly, if at all, crystallizable," and hence he corroborates Durand, and removes the only reason why he hesitated to call his product *hydrastia.* With these explanations of the past history of these principles, we are prepared to accord to Mr. Merrill and Prof. Garrison all credit due to them for their investigations of these principles in the pages which follow, as a valuable contribution to the knowledge of the subject.

that the yellow coloring principle of the xanthorhiza was the same as that of the berberis, but did not suspect their indentity with that of the hydrastis.

To determine this question, we prepared crystallized specimens of the muriate of berberina, and of our hydrastia, and subjected them to a series of comparative experiments, and these *then* led us to the conclusion, that although similar, they were not identical. Among these experiments were the following:

1st. Equal quantities of these muriates were dissolved in a dilute solution of potassa. That of hydrastia remained unchanged in color, whilst that of berberina became hyacinth red, and these colors remained at the end of five days.

2d. Equal portions were dissolved in equal quantities of boiling alcohol. On cooling, the muriate of hydrastia soon formed large stellar crystals, and when cold became semi-solid. The berberina solution when cold, showed only a cloud of extremely fine crystals. And as far as tried, this salt of berberina appeared more soluable in all cold menstrua, than that of the hydrastia.

3. To equal solutions in alcohol, tincture of iodine was added. In the hydrastia solution, large crystals soon appeared, and continued to form until the whole was a sold mass. Those under the microscope appear long, slender, and bright yellow. The berberina solution slowly evolved fine crystals, much less copiously, and these under microscope have a *brownish* hue.

The behavior with iodide of potassium was much the same, and the distinctions similar.

4. Equal solutions were treated with neutral acetate of lead. Both gave copious yellow precipitates, but that of the berberina appeared perfectly amphorous under the microscope (of 150 diam.) while that of the hydrastia was resolved into a mass of silky crystals.

Several other similar tests were applied, some of which showed no appreciable difference between the two salts, while in others differences were discovered that tended to confirm the conclusions drawn from the above, and named in our letter of June, viz.: that the two bases were not identical. We did not resort to ultimate analysis, for want of suitable apparatus.

We have not been able to repeat or extend these experiments for want of suitable material from which to prepare a better specimen of berberina. But we have since prepared the muriate of the alkaloid base of the xanthorhiza, of which we send you a sample labelled, "mur. *xanthia*," and this we have satisfied ourselves is identical with that of the hydrastia, and this fact, together with the analysis of Mahla and Perrins, leads us to believe our experiments above stated to be delusive, in consequence of some imperfection in our preparation of the berberina, of which we had but a small quantity. Hence we admit the conclusion of Mr. Perrins, that the yellow coloring principle of the hydrastis, the berberis, and the xanthorhiza, are in all probability identical.

What then is the appropriate name of this beautiful alkaloid? The hydrastis is by far the most copious source, and that from which it will, no doubt, be chiefly obtained; and on this account should have the preference in giving it its name. Moreover, its salts will, without doubt, become important agents in the materia medica, and it is very desirable on this account that its name should point to the plant from which it is derived. These I think strong arguments, especially the latter, in favor of the name "Hydrastia," which I propose for it. On the other hand, the other alkaloid, which as yet is known to be found in this plant only, must naturally receive its name from it, and the similarity between the names hydrastia and hydrastina may lead to confusion and mistakes. It may also be considered partial to derive from one plant the name for a principle that is found in several. If these objections should be considered paramount, then I suggest for it the name *xanthia*, from the Greek xanthos,—yellow,—a most appropriate name, as it is the only alkaloid known of a bright yellow color.

But although this article is already sufficiently extended, I have not yet reported the processes by which we have obtained these preparations. These are in general indicated in the essays of Mahla and Perrins. But without going over the various experiments we have tried, and the possible processes that may be pursued, I will briefly state those which we have found most simple and elegible.

1st. The ground root may be exhausted, (and this is best done by percolation,) either by alcohol, dilute spirit, or even water.

If it is desired to obtain and preserve the dark resinoid principle which is abundant in the root, and is not without some medicinal value, then alcohol of ordinary strength must be used adding water at the last to drive out the spirit that it may be recovered. The alcohol must then be distilled off, and the resinoid, (which should be named hydrastin,) be allowed to settle and be removed and dried.

2d. To the clear solution, whether obtained by alcohol or water, add hydrochloric acid, as long as a precipitate is formed, or until the liquid is distinctly sour.

This combines with both bases, but the muriate of hydrastia (or xanthia) being sparingly soluable in water, or cold alcohol, is precipitated as a bright yellow powder, and is collected and washed on a filter. A precipitate may be obtained by other acids, and by many salts, especially the muriates, but the most satisfactory results are by free muriatic aid. The impure muriate thus obtained is purified by dissolving it in hot alcohol, and treating it with animal charcoal, as described by others. The clear solution on cooling crystallises in beautiful acicular yellow crystals.

From this all the other salts are readily obtained by processes that will suggest themselves to every chemist. They mostly crystallize in needle-shaped or feathery crystals, and are all of a brilliant yellow color.

A little of the pure base *hydrastia* was obtained by withdrawing the acid from the sulphate of baryta, or what succeeded better in our hands, by oxide of lead. The pure alkaloid is uncrystallizable, but combines readily with the acids which we have presented to it.

The other alkaloid which we had named "hydrastin," is still in the mother liquor. From this it is precipitated in its basic condition by an alkali, say soda of ammonia, which combines with its organic acid, or the hydrochloric acid with which it is now combined, and let it fall as a greyish white powder, as it is wholly insoluable in cold aqueous fluids. This is purified by repeated solution in boiling alcohol, and crystallization from it on cooling. It crystallizes in large, quadrangular prisms, with very acute parymidal summits, and when pure are white or colorless. Although itself is quite insoluable in water, and is easily crystallized, it readily combines with all the acids, and forms salts that are very soluable, and difficultly, if at all, crystallizable: presenting in these respects exactly the reverse of the other alkaloid.

When the preservation of the resinoid principle is not desired, these alkaloids may be more economically obtained by exhausting the ground root with dilute sulphuric acid, instead of alcohol, as the sulphates of both of them are readily soluable, but the process is somewhat more circuitous and complicated, and I will not extend this already too long paper to describe it.

We have isolated the organic acid with which these bases are combined in their normal state, but not in sufficient quantity or purity to enable us to determine whether it be a distinct principle, or is identical with some of the vegetable acids already known. If time permits we will investigate the matter farther.

Submitting these remarks, I remain, yours truly,

WM. S. MERRILL, A.M.

*Cincinnati, August 25th,* 1862.

